# Supplementary material for: Smoking cessation and counseling: A mixed methods study of pediatricians and parents
Source: PLoS One. 2021 Feb 9;16(2):e0246231. doi: 10.1371/journal.pone.0246231 (PMC7872228; doi:10.1371/journal.pone.0246231)
Supplement: S1 File — This survey was distributed to all families of children and youth seeking care at their pediatrician’s office. (DOCX) [file pone.0246231.s001.docx]

**S1: Family Smoking Survey**

The Easy Breathing Program at Connecticut Children’s Medical Center, the CT American Academy of Pediatrics and the Tobacco Control Program at the State DPH in partnership with your child’s pediatrician are conducting a survey to better understand how to prevent youth smoking and exposure to second hand smoke. Your participation is voluntary and anonymous.

1. Do you or anyone who lives in your household currently smoke cigarettes? YES______ NO _____
2. Do you or anyone who lives in your household currently use/vape electronic cigarettes? YES______ NO _____

If “**no” to both questions**, thank you for your time. Please place your survey in the marked box.

3. If “**yes” to smoking cigarettes**,

a. How long have you smoked cigarettes? ___________(circle one) months or years

b. How many cigarettes/day on average do you smoke? ____________(circle one) number or packs/day

4. If “**yes” to vaping (using electronic cigarettes)**

a. How long have you vaped? ________________(circle one) months or years

b. How may ampoules/vials on average do you vape each day?___________number/day

5. Do you currently smoke or vape inside the car? YES______ NO _____

inside your home? YES______ NO _____

6. During the past six months, have you tried to quit smoking cigarettes or electronic cigarettes? YES___ NO ____

5a. If “yes”, how did you try to quit? Check all that apply

- Cold turkey
- Used smoking cessation aides (not including e-cigarettes or vaping)
- Attended a smoking cessation program
- Partnered with another individual who wanted to quit
- Used e-cigarettes
- Other (explain)________________________________________

7. How interested are you in quitting smoking/vaping? (Circle one)

Not interested A little interested Somewhat interested Interested Very interested

8. If you are at all interested in quitting smoking, what is the best way to get information about how to quit (check all that apply) to you

- Quit line (telephone counseling)
- Physician’s office
- Phone app
- Brochure
- Other: (explain) _________________________

9. What is your race (Choose one):

- Hispanic/ Puerto Rican
- Hispanic/Central or South America
- Hispanic/Other
- Not Hispanic

10. What is your ethnicity (Choose one):

- Caucasian
- African American
- Caribbean/Virgin Island
- Other

11. What is the zip code where you live?______________ 12. What is your age in years? _______

Thank you for helping up to better understand how we can partner with families around smoking prevention and cessation. Please put your survey in the marked box.
