# Supplementary material for: Smoking cessation and counseling: A mixed methods study of pediatricians and parents
Source: PLoS One. 2021 Feb 9;16(2):e0246231. doi: 10.1371/journal.pone.0246231 (PMC7872228; doi:10.1371/journal.pone.0246231)
Supplement: S2 File — This survey was distributed to all pediatricians participating in the Easy Breathing asthma management program across Connecticut. (DOCX) [file pone.0246231.s002.docx]

**S2: Clinician Smoking Survey**

**Easy Breathing, the CT American Academy of Pediatrics and the Tobacco Control Program at DPH are trying to learn more about how we can help pediatric clinicians provide smoking cessation/prevention information to families. Could you please complete this anonymous survey and return it to your Easy Breathing coordinator.**

1. How often do you ask your families about their smoking behaviors at home?

Never One time (first visit) Yearly >yearly<every visit Every visit

1. What smoking cessation services do you offer parents of your patients who smoke? Check all

- Educational materials
- Referral to smoking cessation programs
- Materials on smoking cessation aids
- Other__________________________________________________
- I do not provide any smoking cessation services for parents

1. At what age do you start counseling your patients about cigarette smoking? _____Years
2. How often do you counsel adolescents about the dangers of smoking cigarettes?

Never One time (first visit) Yearly >yearly<every visit Every visit

1. How often do you counsel adolescents who smoke about the dangers of electronic cigarettes?

Never One time (first visit) Yearly >yearly<every visit Every visit

1. How often do you counsel your adolescent patients who do not smoke about the dangers of electronic cigarettes?

Never One time (first visit) Yearly >yearly<every visit Every visit

1. How effective is what you do with your patients and families related to smoking cessation and prevention?

Not effective Slightly effective Somewhat effective Effective Very effective

1. How confident are in counseling parents about smoking cessation?

Very confident Confident Somewhat confident Slightly confident Not confident

1. How confident are you in counseling adolescents against smoking initiation?

Very confident Confident Somewhat confident Slightly confident Not confident

1. Relative to cigarettes, how dangerous or hazardous to health are electronic cigarettes?

Not as dangerous Equally dangerous More dangerous

1. How much does reimbursement for counseling services influence your smoking counseling activities?

Big role Medium Role Small role No role at all

1. I know how to code for tobacco related counseling services. True False

Thank you for your help with this. We will provide the results in the aggregate and by practice and we will use this information to develop smoking prevention/cessation programs that fit into your work flow.
