# Supplementary material for: Smoking cessation and counseling: A mixed methods study of pediatricians and parents
Source: PLoS One. 2021 Feb 9;16(2):e0246231. doi: 10.1371/journal.pone.0246231 (PMC7872228; doi:10.1371/journal.pone.0246231)
Supplement: S3 File — This focus group guide was used to facilitate discussions with pediatricians regarding their approach to somking prevention and cessation among their patients and families. (DOCX) [file pone.0246231.s003.docx]

Today’s Date: / /

(Month) (Day) (Year)

**S3: Primary Care Focus Group Guide**

Site: _____________

Number of participants: ______________________________

Focus Group Leader: ____________________

Focus Group Notetaker: ________________________

**Introduction**

Good afternoon and welcome. Thank you for coming to our focus group for the primary care community. My name is _______________and I am from the Asthma Center at Connecticut Children’s Medical Center. I will be leading our meeting this afternoon. Assisting me is____________, also from the Asthma Center.

We are interested in learning more about what clinicians currently do and want to do regarding asthma in general and smoking prevention and cessation in children with asthma and their families. We also want to learn about how you view Community Health Workers as part of the health care team especially for children with asthma. This information will then be used to develop an asthma program that is composed of multiple layers and multiple activities to create a comprehensive sustainable system of care for children with asthma here in Hartford.

This study is being conducted by the Connecticut Children’s Medical Center and the University of Connecticut. Dr. Michelle Cloutier is the principal investigator. Our meeting this afternoon will be approximately 90 minutes long. Please help yourself to the food.

I know most of you have participated in focus groups before but in case there are some newcomers, here is what is going to happen. I am here to ask some questions, encourage everyone to share, and give everyone an opportunity to speak. We would like to ask that everyone participate in the discussion. There are no right or wrong answers. We are hoping to listen as much as possible. So, if you contribute a lot, I may ask you to give others a chance to respond. And if you are holding back, I may ask you directly what your thoughts are. It is OK if your opinion is different than someone else’s opinion. Please feel free to share your point of view even if it differs from what others have said. We just want to make sure all of you have a chance to share.

We will be recording this session because we don’t want to miss any of your comments. Your confidentiality will be protected—there are no names on the report and all of your comments are confidential.

Each of you has a number—we will use these to refer to each other throughout the conversation.

Lastly, please do not use your cell phones or text during the session.

Now, to get things started would like each of you to complete a brief questionnaire. You will not be identified and these results will only be presented in the collective.

**Part I: Smoking**

**Introduction**: Smoking and environmental tobacco smoke exposure is a major problem for children with asthma in Hartford. Is there anyone who does not agree with this statement?

**Question 1:** What types of programs currently exist in Hartford for smoking cessation in adults?

1a. Do you ever refer parents to smoking cessation programs?

If not, why not?

2a. Do you ever refer adolescents to smoking cessation programs?

**Question 2**: What types of programs or materials do you think would be helpful for pediatricians to be aware of to help parents to quit smoking?

Educational materials

What are the most effective messages?

Tailored materials?

Communication with parent’s primary care clinician?

What types of materials do you currently use?

What types of materials would you like to use?

Videos in the waiting room?

**Question 3:** There are 3 groups of smoking cessation materials and programs currently available in Hartford

Quit line

Smoking cessation classes

Web-based programs

Smart phone apps

**Question 4**: What strategies do you think would be most effective to prevent youth from initiating smoking?

Educational materials

What are the most effective messages?

Tailored materials

What should these look like?

**Question 5:** What do you need as a clinician to help you to counsel

Families?

Adolescents?

Younger Children?

Moderator briefly summarizes the discussion

**Question 6**: Is there anything else you would like to say or add before moving on to the next section?
