# Supplementary material for: Smoking cessation and counseling: A mixed methods study of pediatricians and parents
Source: PLoS One. 2021 Feb 9;16(2):e0246231. doi: 10.1371/journal.pone.0246231 (PMC7872228; doi:10.1371/journal.pone.0246231)
Supplement: Attachment — Submitted filename: Response to Reviewers_2.docx [file pone.0246231.s005.docx]

Response to Reviewers:

The manuscript mentions clinicians wanting more resources to help parents and teens quit smoking. My one final, emphatic suggestion would be to mention by name the AAP's Richmond Center: [https://www.aap.org/en-us/advocacy-and-policy/aap-health-initiatives/Richmond-Center/Pages/default.aspx](https://urldefense.proofpoint.com/v2/url?u=https-3A__www.aap.org_en-2Dus_advocacy-2Dand-2Dpolicy_aap-2Dhealth-2Dinitiatives_Richmond-2DCenter_Pages_default.aspx&d=DwMGaQ&c=qS4goWBT7poplM69zy_3xhKwEW14JZMSdioCoppxeFU&r=EaKLuGHeSk1h8VIf5LJqo7x1mcRQo3Alrc_5IyRS0nII1T8bNsCxZowi8mrQ7DjX&m=o6vUTxt69Bm9vfH3i27q1GvidzNGNXHYZFvwLmPMBKc&s=Lyt8J2FPBJacSfyyMvaHZwFJdwKOk4VByHgIfqHiF3g&e=). The Richmond Center has so many great practical resources for pediatricians, and yet far too few pediatricians are aware of this up-to-date, well-curated resource.

*Thank you for this recommendation. We have added this resource to the discussion, page 16, line 334. We agree, it has a plethora of practical resources!*

Few comments for further consideration:
1. The authors should consider adding the calculation of odds ratio and 95% confidence intervals, rather than just showing the p values which does not indicate the direction of association.

*We attempted to calculate odds ratios and 95% confidence intervals. Unfortunately, due to our small numbers with the vaping data, this resulted in some empty cells (see alternative Table 2 below). Given this, we felt that our original table with p values was more appropriate for our data.*

| Table 2. Association of Demographic and Smoking/Vaping Characteristics With Interest in Quitting Smoking/Vaping | | | |
| --- | --- | --- | --- |
|  | Uninterested | Interested | Unadjusted Model |
|  | n (%) | n (%) | OR (95% CI) |
| **Smokers** |  |  |  |
| Cigarettes smoked per day (n=170) |  |  |  |
| 1-9 | 17 (37.8) | 51 (40.8) | 1 [Reference] |
| 10-19 | 13 (28.9) | 43 (34.4) | 1.28 (0.60-2.74) |
| ≥ 20 | 15 (32.6) | 31 (24.8) | 0.80 (0.37-1.70) |
| Years smoked (n=159) |  |  |  |
| 1-9 | 15 (34.9) | 39 (33.6) | 1 [Reference] |
| 10-19 | 18 (41.9) | 42 (36.2) | 0.94 (0.46-1.91) |
| ≥ 20 | 10 (23.3) | 35 (30.2) | 1.12 (0.85-1.48) |
| Residence by 5 CT’s (n=193) |  |  |  |
| Suburban | 5 (9.4) | 16 (11.4) | 1 [Reference] |
| Rural | 4 (7.5) | 14 (10.0) | 1.23 (0.30-5.00) |
| Urban periphery | 22 (41.5) | 52 (37.1) | 0.83 (0.31-2.24) |
| Urban core | 22 (41.5) | 58 (41.4) | 0.92 (0.34-2.49) |
| Race/ethnicity (n=190) |  |  |  |
| Non-Hispanic white | 30 (58.8) | 70 (50.4) | 1 [Reference] |
| Hispanic | 15 (29.4) | 53 (38.1) | 1.60 (0.79-3.23) |
| Non-Hispanic black or other | 6 (11.7) | 16 (11.5) | 1.21 (0.44-3.36) |
| **Vapers^c^** |  |  |  |
| Ampoules/vials vaped per day (n=17) |  |  |  |
| <1 | 3 (50.0) | 0 (0.0) | 1 [Reference] |
| ≥ 1 | 3 (50.0) | 10 (100.0) | N/A |
| Number of years vaped (n=27) |  |  |  |
| 0-1 | 7 (77.8) | 16 (88.9) | 1 [Reference] |
| ≥ 2 | 2 (22.2) | 2 (11.1) | 0.44 (0.05-3.76) |
| Residence by 5 CT’s (n=40) |  |  |  |
| Suburban | 2 (16.7) | 4 (14.3) | 1 [Reference] |
| Rural | 1 (8.3) | 8 (28.6) | 4.0 (0.27-58.56) |
| Urban periphery | 5 (41.7) | 13 (46.4) | 1.30 (0.18-9.47) |
| Urban core | 4 (33.3) | 3 (10.7) | 0.38 (0.04-3.61) |
| Race/ethnicity (n=39) |  |  |  |
| Non-Hispanic white | 7 (58.3) | 18 (66.7) | 1 [Reference] |
| Hispanic | 3 (25.0) | 5 (18.5) | 0.61 (0.12-3.27) |
| Non-Hispanic black or other | 2 (16.6) | 4 (14.8) | 0.74 (0.11-4.96) |
| Abbreviations: OR, Odds Ratio; CI, Confidence Interval; N/A; Not Applicable | | | |

*If you feel otherwise and would like us to use this table instead of the one in the manuscript, please let me know. It does not change the text.*

2. Authors should also include harm reduction role of vaping while discussing the findings

*This is an important point and we have added mention of this to the discussion on page 16, line 327.*
