# Supplementary material for: Smoking cessation and counseling: A mixed methods study of pediatricians and parents
Source: PLoS One. 2021 Feb 9;16(2):e0246231. doi: 10.1371/journal.pone.0246231 (PMC7872228; doi:10.1371/journal.pone.0246231)
Supplement: Attachment — Submitted filename: Response to Reviewers_3.docx [file pone.0246231.s006.docx]

Response to Reviewers:

Please also report odds ratios and 95% confidence intervals, rather than just showing the p values which does not indicate the direction of association

We have now replaced Table 2 to show odds ratios and 95% confidence intervals and made the necessary changes in the abstract and results.
